# Symptom clusters and their temporal patterns in breast cancer patients undergoing chemotherapy: a systematic review of cross-sectional and longitudinal studies

**DOI:** 10.3389/fonc.2026.1717693

**Published:** 2026-03-16

**Authors:** Liyao Su, Lin Qiu, Tongping Gu, Yongmei Jin

**Affiliations:** 1 Department of Nursing, Seventh People’s Hospital of Shanghai University of Traditional Chinese Medicine, Shanghai, China; 2 School of Nursing, Shanghai University of Traditional Chinese Medicine, Shanghai, China; 3 Graduate School, Dali University, Yunnan, China

**Keywords:** breast cancer, chemotherapy, longitudinal symptom trajectories, oncology nursing, symptom clusters, systematic review

## Abstract

**Background:**

Patients with breast cancer undergoing chemotherapy commonly experience multiple co-occurring symptoms, which often cluster together to form symptom clusters, characterized by temporal variability and a significant clinical burden.

**Objective:**

The aim of this systematic review was to identify common symptom clusters in breast cancer patients during chemotherapy, to examine their temporal development during the different treatment phases, and to summarize the implications for personalized symptom management in clinical nursing practice.

**Methods:**

In line with the PRISMA guidelines, a systematic literature search was carried out in eight databases covering studies published up to 25 September 2025. Eligible studies were required to identify clusters of symptoms using statistical methods. Both cross-sectional and longitudinal studies have been undertaken. The selection of the study, data extraction and quality assessment were carried out independently by two evaluators.

**Results:**

A total of 24 studies (N = 5,234 patients) were enrolled. Thirteen different symptom clusters were identified, most frequently involving gastrointestinal, psychological, neurological, and hormone-related symptoms. The composition and severity of the clusters varied between the stages of chemotherapy. Gastrointestinal clusters were highest in the early treatment cycles, while fatigue, cognitive, and psychosocial clusters persisted or intensified in later or post-chemotherapy phases. Evidence from longitudinal studies suggested that symptom patterns evolve and clusters reconfigure over time.

**Conclusions:**

Symptom clusters in breast cancer patients receiving chemotherapy are heterogeneous and temporally dynamic. Strict symptom assessment and tailor-made interventions tailored to the phase of chemotherapy can help to reduce the burden of symptoms and improve patient-centered care. Future studies should standardize time point definitions, adopt longitudinal design and incorporate culturally representative samples to advance the science of oncology symptoms.

**Systematic Review Registration:**

https://www.crd.york.ac.uk/PROSPERO/display_record.php?ID=CRD42025638742, identifier CRD42025638742

## 1 Introduction

It is estimated that approximately 2.3 million new cases of breast cancer occurred globally in 2023, making it the most prevalent malignant tumor among women (1). Due to its high incidence and mortality rates, breast cancer has emerged as a critical public health challenge with significant implications for women’s health worldwide (2). With continuous advances in medical technology, chemotherapy has become an essential component of comprehensive breast cancer treatment. Evidence indicates that chemotherapy can effectively inhibit tumor growth and eliminate potential micrometastases, thereby significantly improving patient survival rates (3–5). However, while chemotherapy confers significant therapeutic benefits, it is frequently associated with substantial adverse effects. Chemotherapy drugs affect not only cancer cells but also damage normal cells and tissues. Patients commonly experience a constellation of physiological and psychological symptoms, including pain, nausea, vomiting, anorexia, fatigue, immunosuppression, and neurological damage (6, 7). Typically, these adverse reactions do not exist in isolation but rather interact and mutually exacerbate each other, further increasing patients’ overall symptom burden and severely affecting their quality of life and treatment adherence.

Traditional research has predominantly focused on the analysis of individual symptoms, yet this approach fails to capture the full complexity of patients’ lived experiences. In recent years, researchers have gradually focused on a more holistic form of symptom presentation: symptom clusters. Symptom clusters refer to collections of two or more symptoms that co-occur temporally and have intrinsic correlations statistically or clinically. These clustered symptoms may originate from common pathophysiological mechanisms, treatment side effects, or similar psychological reactions (8, 9). Therefore, identifying and dynamically tracking symptom clusters is of great significance for improving clinical care quality and optimizing intervention strategies. Among breast cancer patients undergoing chemotherapy, common symptom clusters include gastrointestinal symptom clusters (such as nausea, vomiting, decreased appetite), psychological symptom clusters (such as anxiety, depression, insomnia), and cognitive dysfunction clusters (such as memory decline, attention deficits). The composition and manifestation of these symptom clusters may change with different chemotherapy cycles, presenting certain dynamic evolutionary characteristics.

Although an increasing number of studies have focused on symptom cluster phenomena during breast cancer chemotherapy, the current literature has the following major limitations: First, most studies adopt cross-sectional designs that can only reflect symptom co-occurrence at specific time points and fail to reveal the changing trends of symptom clusters throughout the chemotherapy process. Chongkham et al. identified distinct symptom clusters on the seventh day following chemotherapy but did not further explore their evolutionary patterns in subsequent cycles (10). Second, some studies have limited sample sizes and are mostly concentrated in single healthcare institutions or regions, limiting the external generalizability of their findings. Therefore, there is an urgent need for a more systematic and comprehensive review of symptom clusters in breast cancer patients undergoing chemotherapy, while paying attention to their potential temporal changes.

Because symptom clusters evolve across chemotherapy cycles, with their composition, intensity, and co-occurrence patterns changing over time, a longitudinal, whole-trajectory perspective is essential to capture their dynamic trajectories and inform stage-tailored symptom management. In response to this research gap, this article presents a systematic review of existing literature on symptom clusters in breast cancer patients undergoing chemotherapy, integrating evidence from both cross-sectional and longitudinal studies to elucidate the dynamic trajectories of symptom clusters across treatment cycles. We further recommend standardizing symptom cluster reporting across fixed trajectory anchors-early stage (first cycle, within 1–2 weeks post-infusion), middle stage (second-third cycles, approximately weeks 3-6), late stage (≥ 6 weeks or after the fourth cycle), and post-chemotherapy follow-up, to improve comparability across studies and facilitate trajectory-based symptom management. This review aims to identify and synthesize commonly reported symptom clusters during chemotherapy and their characteristic compositions. It further seeks to characterize the evolution of these symptom clusters across treatment cycles, highlighting temporal patterns and trends based on available evidence. Finally, the review summarizes clinical implications for nursing practice, proposes feasible and stratified intervention strategies, and discusses existing research limitations and areas requiring further investigation.

## 2 Method

### 2.1 Design

The methodological framework for this study is based on the Preferred Reporting Items for Systematic Reviews and Meta-Analyses (PRISMA) guidelines. No changes were made to the information given after registration, and this review has been documented in PROSPERO (No. CRD42025638742).

### 2.2 Inclusion and exclusion criteria

This study established eligibility criteria based on the PEO framework. Population: breast cancer patients aged ≥ 18 years undergoing chemotherapy, covering all stages before, during, and after chemotherapy, without limiting their healthcare settings; Exposure: patients must receive chemotherapy treatment of any regimen or cycle, with no restrictions on specific chemotherapy drugs or protocols. Outcome: studies must have identified symptom clusters using statistical methods such as factor analysis, principal component analysis, cluster analysis, latent class or transition analysis, and must report their composition, prevalence or severity, temporal patterns, and clinical associations.

Regarding study types, the review includes cross-sectional or longitudinal quantitative studies, including original survey research and secondary data studies, while excluding case reports, case series with insufficient sample sizes (n < 10), intervention studies not involving symptom cluster identification, as well as reviews, commentaries, conference abstracts, and qualitative studies. The literature search time range was set from database inception to September 25, 2025, with language restrictions to Chinese and English published literature, excluding literature in other languages. Additionally, all included studies must pass quality assessment using the standard quality assessment tool developed by Kmet et al., with studies scoring below 50% being excluded (detailed methods for quality assessment are described in Section 2.6).

### 2.3 Search strategy

Searches were conducted in PubMed, Web of Science, Embase, the Cochrane Library, CNKI, VIP Database, Wanfang Data, and the China Biomedical Literature Database, with the search time extended to September 25, 2025. The English search terms consist of subject terms and free words. Taking PubMed as an example: ((((((((((“Breast Neoplasms”[Mesh]) OR (Breast Neoplasm[Title/Abstract])) OR (Breast Tumor[Title/Abstract])) OR (Breast Cancer[Title/Abstract])) OR (Cancer of Breast[Title/Abstract])) OR (Malignant Neoplasm of Breast[Title/Abstract])) OR (Mammary Cancer[Title/Abstract])) OR (Mammary Neoplasm, Human[Title/Abstract])) OR (Breast Carcinoma[Title/Abstract])) OR (Mammary Carcinoma, Human[Title/Abstract])) AND ((((((“Syndrome”[Mesh]) OR (Symptom Cluster[Title/Abstract])) OR (Symptom constellation[Title/Abstract])) OR (Concurrent symptom[Title/Abstract])) OR (multiple symptom[Title/Abstract])) OR (symptom combination[Title/Abstract])). Search strategies for other databases are detailed in **Appendix 1**. Additionally, to improve coverage, we also reviewed the reference lists of included studies to identify supplementary studies.

### 2.4 Study selection

**Figure 1:**
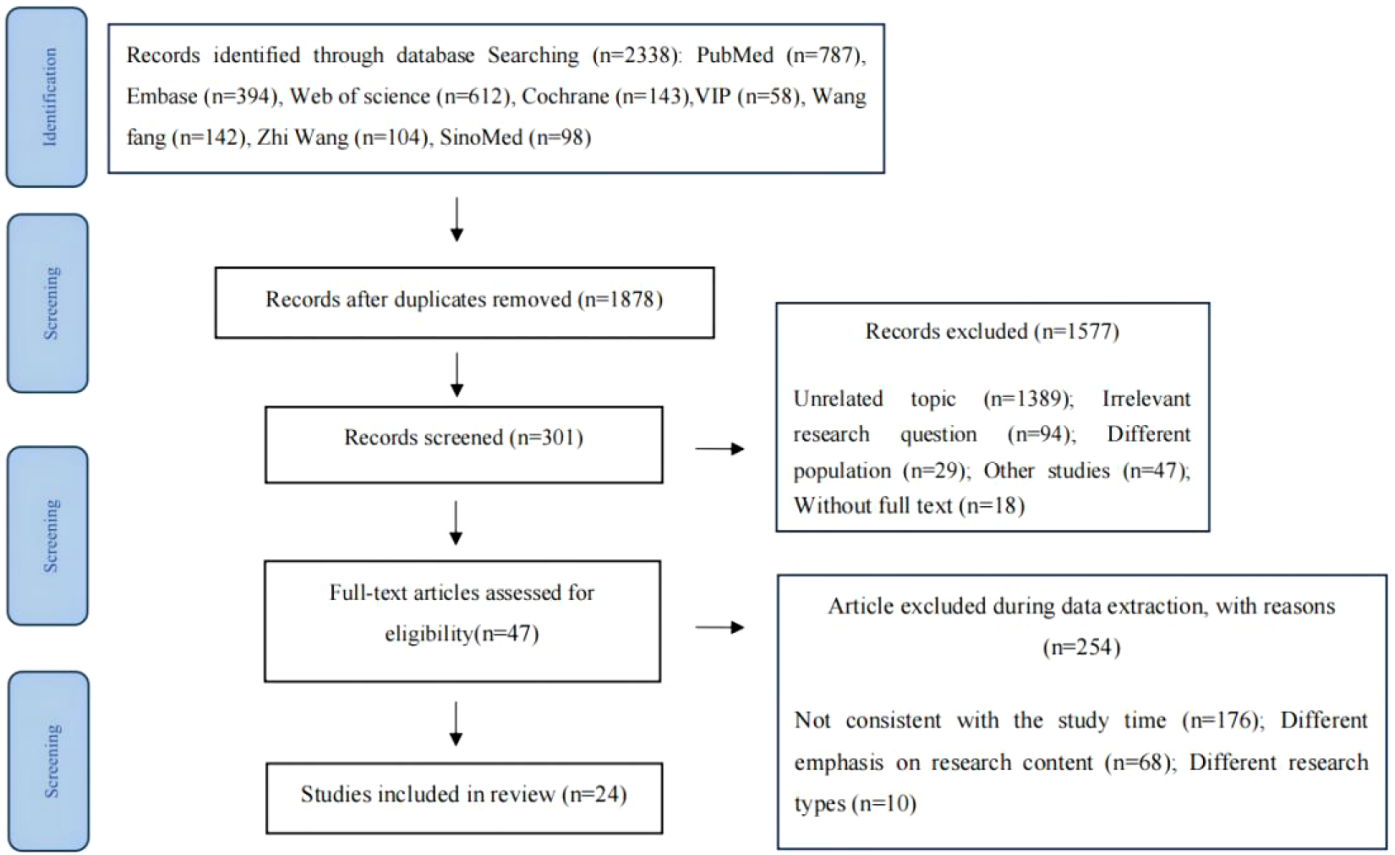
PRISMA flow diagram.

The acquired material was imported into EndNote X9, and duplicate entries were removed. Two postgraduate nursing students proficient in evidence-based medicine independently screened the titles and abstracts for the initial screening, and then conducted a second round of screening by reviewing the full texts to ensure consistency, achieving an inter-rater agreement of 90% (Cohen’sκ=0.85). A third reviewer resolved disagreements. The final number of included studies and exclusion reasons are presented in the form of a PRISMA 2020 flow chart (see **Figure 1**).

### 2.5 Data extraction

During data extraction, we recorded whether each included study reported ethics committee/institutional review board (IRB) approval and/or informed consent procedures, where applicable. Studies that did not clearly report ethics-related information were noted as “not reported”. Two researchers independently extracted data from the included literature, and they discussed any disagreements with the third researcher. **Appendix 2** provides precise information on the extracted data, which included the first author, country, study type, sample size, cancer stage, and chemotherapy medications. Evaluation time, assessment tools/dimensions, analytic techniques, symptom cluster types, etc., were among the details about symptom clusters. A summary analysis was carried out; see **Table 1** for more details.

**Table 1:** Extracted information on symptom clusters from included studies.

Author	Assessment tools/dimensions	Analytic techniques/inclusion criteria	Evaluate time	Symptom cluster types
Chongkham-Ang et al (10)	MSAS/Severity/Degree of distress	Principal component analysis/exploratory factor analysis	Seven days after chemotherapy	①②③⑧⑨
Vuttanon et al (11)	ESAS/Severity	exploratory factor analysis	Post-chemotherapy	①②⑥⑧
Langford et al (12)	Single symptom assessment Scale/Severity	Profiling of potential classes	Postoperative chemotherapy cycle≥ one time	①⑥⑧
Deng et al (13)	Symptom assessment scale for breast cancer patients undergoing chemotherapy	Exploratory factor analysis/symptom rate > 10%	Postoperative chemotherapy cycle≥ two times	①②③④
Jiang et al (14)	MDASI	Exploratory factor analysis	Postoperative chemotherapy	①②
Li et al (15)	Symptom assessment scale for breast cancer patients undergoing chemotherapy	Exploratory factor analysis/symptom rate > 20%	Chemotherapy was performed 1 times, 2 times, 3 times, or ≥4 times	①②④⑦
Li et al (16)	CD-RISC、MDASI	Principal component analysis/exploratory factor analysis	Postoperative chemotherapy	①②⑤
Liu et al (17)	MDASI	Exploratory factor analysis	Postoperative chemotherapy cycle≥ one time	①②⑤⑥
Wang et al (18)	Symptom assessment scale for breast cancer patients undergoing chemotherapy/Severity	Exploratory factor analysis/symptom rate > 10%	Postoperative chemotherapy	①④⑤⑦
Zhang et al (19)	MSAS	Exploratory factor analysis/symptom rate > 20%	Postoperative chemotherapy cycle≥ two times	①②③④
Liang et al (20)	MDASI	Profiling of potential classes	Before chemotherapy, after the first chemotherapy, after the third chemotherapy, after the last chemotherapy	①②⑤
Zhou et al (21)	Symptom assessment scale for breast cancer patients undergoing chemotherapy	Correlation analysis/comparative analysis	Postoperative chemotherapy cycle≥ one time	②④⑤⑦
Albusoul et al (22)	HADS、SES/Severity	Exploratory factor analysis/symptom rate > 20%	2 days before chemotherapy(T1), 7 days after the 3rd chemotherapy(T2),7 days after the 4th chemotherapy(T3)	②⑩
			30 days after chemotherapy (T4)	⑩
Browall et al (23)	MSAS	Principal component analysis/mean symptom score > 0.5	Baseline (T1), 1 month of chemotherapy (T2), 3 months of chemotherapy (T3), 6 months of chemotherapy (T4)	①②⑬
Sullivan et al (24)	MSAS/Incidence/Severity	Exploratory factor analysis	1 week before chemotherapy (T1)	①②④⑦⑪⑫
			1 week after chemotherapy (T2), 2 weeks after chemotherapy (T3)	①②④⑦⑨
Golan-Vered et al (25)	Single symptom assessment Scale/Severity	Cluster analysis	Before chemotherapy (T1), after at least 2 cycles (T2)	①⑥⑧
Berger et al (26)	HADS、SES/Severity	Exploratory factor analysis/symptom rate > 20%	Before the first adjuvant chemotherapy (T1), 1 month after the last chemotherapy (T2), 1 year after baseline (T3)	②⑩
Sanford et al (27)	Single symptom assessment Scale/Severity	Cluster analysis/mixed effect model analysis	Before chemotherapy (T1), 7 days before the 4th cycle of chemotherapy (T2), 6 months after the start of chemotherapy (T3)	⑤⑥
Huang et al (28)	SCL-90、TRSC	Correlation analysis	Completion of the first cycle of chemotherapy one day before the second cycle of chemotherapy (T1), one day before the fourth cycle of chemotherapy (T2), one month after the end of chemotherapy (T3)	②④⑤⑦
Li et al (29)	MSAS、FACT-B	Correlation analysis	Pre-chemotherapy (T1)	①⑧
			After the first chemotherapy (T2)	①②③④⑧
			After the third chemotherapy (T3), after the sixth chemotherapy (T4)	①②③④⑤⑧
Zhu et al (30)	MSAS	Exploratory factor analysis/symptom rate > 20%	Pre-chemotherapy 2d (T1)	①③④
			1 week after the first chemotherapy (T2), 1 week after the third chemotherapy (T3)	①②③④⑤
Luo et al (31)	MSAS	Exploratory factor analysis/symptom rate > 20%	The first chemotherapy (T1)	①②④
			Second chemotherapy (T2), third chemotherapy (T3)~ seventh chemotherapy (T7)	①②③④⑤
			eighth chemotherapy (T8)	①②④⑤
Hsin-Tien Hsu et al (32)	MDASI	Potential category growth analysis	21 consecutive days after the third cycle of chemotherapy	①②⑤⑥
Wang et al (33)	MDASI	Exploratory factor analysis/symptom rate > 20%	Chemotherapy Week 1 (T1)	①⑧
			1st month of chemotherapy (T2), 3rd month of chemotherapy (T3)	①③④⑤⑧

MSAS refers to the Memorial Symptom Assessment Scale, ESAS to the Edmonton Symptom Assessment Scale, MDASI to the Anderson Symptom Assessment Scale, CD-RISC to the Cancer Treatment Functional Assessment Scale-Fatigue, HADS to the Hospital Anxiety and Depression Scale, SES to the Symptom Experience Scale, SCL-90 to the Symptom Checklist-90, TRSC to the Williams Chinese Version Treatment-Related Symptom List, and FACT-B to the Breast Cancer Treatment Functional Evaluation System. In the symptom clusters, ①represents the psychological symptom cluster, ②the gastrointestinal symptom cluster, ③the self-image disturbance symptom cluster, ④the hormone-related symptom cluster, ⑤the neurologic symptom cluster, ⑥the fatigue-related symptom cluster, ⑦the skin and mucosa symptom cluster, ⑧the pain-related symptom cluster, ⑨the nutritional symptom cluster, ⑩the treatment-related symptom cluster, ⑪ the illness behavior symptom cluster, ⑫the postoperative symptom cluster, and ⑬the physical symptom cluster.

### 2.6 Quality assessment

The quality of study reporting in the included studies was evaluated using the Standard Quality Assessment Criteria for Evaluating Primary Research Papers from a Variety of Fields, developed by Kmet et al. This quality assessment tool, which has been widely used for appraising primary research in systematic reviews, is particularly useful in observational study contexts. **Appendix 3** lists the items used for assessing the quality of the studies. The 14-item checklist evaluated study design, sample representativeness, data validity, and statistical rigor. Items were scored as ‘YES’ = 2, ‘PARTIAL’ = 1, ‘NO’ = 0. Items that are not applicable are marked with “N/A” and are not scored. The total score is calculated by summing the scores of all appropriate entries and presented as a percentage score. The quality of the assessed studies was classified as limited (< 50%), adequate (50%-70%), good (70%-80%), and strong (> 80%). Studies with a quality score below 50% were excluded from the review. All the scores can be found in **Appendix 4**. One reviewer initially conducted the quality assessment, and a second reviewer independently verified the results. Any disagreements between the two reviewers were resolved through discussion.

### 2.7 Temporal analysis of symptom clusters

To examine the stability and evolutionary trends of symptom clusters reported in the literature during chemotherapy, we extracted symptom cluster compositions at various time points from longitudinal studies and conducted comparative analyses to examine changes over time. According to established criteria in the literature, a symptom cluster is considered relatively stable if it retains at least 75% of its core symptoms across different treatment stages. Due to heterogeneity among the included studies in terms of patient characteristics and statistical methods, and because some studies did not provide sufficiently comparable data, quantitative meta-analysis was not performed. The relevant results are presented in the form of a narrative synthesis.

### 2.8 Data synthesis

This study employed a narrative synthesis strategy to categorically summarize the results of cross-sectional and longitudinal studies, with classification based on research stages, cluster types, statistical methods, and other factors. Particular attention was paid to the compositional stability of symptom clusters, timing of occurrence, and clinical associations. When study reports provided data from multiple time points, we attempted to construct cluster evolution diagrams according to temporal sequence to reveal the potential formation pathways and evolutionary trajectories of symptom clusters.

## 3 Results

### 3.1 Search results

A total of 3,516 articles were initially identified from eight databases. After removing duplicates, 3,208 records remained. Following title and abstract screening and full-text review, 24 studies were included in the final analysis (13 in English (10–12, 20, 22–27, 31–33), 11 in Chinese (13–19, 21, 28–30)). The selection process is illustrated in **Figure 1**, and study characteristics are summarized in **Appendix 1**.

### 3.2 Quality assessment of the included studies

Quality assessment was conducted using the Standard Quality Assessment Criteria developed by Kmet et al. As some items (specifically items 5–7 and 9) are only applicable to RCTs and item 12 is not suitable for descriptive or exploratory designs, these were marked as not applicable (N/A) where appropriate, consistent with the tool’s guidelines.

Among the 24 included studies, quality scores ranged from 11 to 17, all exceeding the 50% threshold. Therefore, no studies were excluded based on quality concerns. According to the scoring classification, one study was rated as adequate (14, 28), seven as good (11, 15–17, 21, 25, 29), and the remaining fifteen studies were rated as strong (10, 12, 13, 18–20, 22–24, 26, 27, 30–33). Detailed scoring results are provided in **Appendix 4**.

### 3.3 Study characteristics

The included studies were published between 2013 and 2024, conducted across China (n = 15) (13–21, 28–33), the United States (n = 5) (12, 22, 24, 26, 27), Thailand (n = 2) (10, 11), Sweden (n = 1) (23), and Israel (n = 1) (25). Among them, 12 adopted cross-sectional designs (10–21) and 12 were longitudinal (22–33), covering the full trajectory from pre- to post-chemotherapy.

Chemotherapy regimens varied, with 16 studies reporting specific protocols and 8 not detailing regimens. Assessment tools commonly used included the MSAS (n = 7) (10, 19, 23, 24, 29–31), MDASI (n = 6) (14, 16, 17, 20, 32, 33), FACT-B (n = 1) (29), and HADS (n = 2) (22, 26), among others (see **Table 1**).

Symptom cluster identification methods varied: exploratory factor analysis was most common (n = 15) (10, 11, 13–19, 22, 24, 26, 30, 31, 33), followed by principal component analysis (n = 3) (10, 16, 23), latent profile/class analysis (n = 3) (12, 20, 32), cluster analysis (n = 2) (25, 27), and correlation-based methods (n = 3) (21, 28, 29). Symptom incidence rates ranged from 10% to over 20%, though several studies did not report exact values.

### 3.4 Symptom cluster characteristics

Across the 24 included studies, thirteen distinct symptom clusters were identified. The most commonly reported clusters involved gastrointestinal, psychological, neurological, and hormone-related symptoms. In contrast, clusters associated with pain, fatigue, skin and mucosal manifestations, self-image disturbance, and social functioning were less frequently documented.

To ensure conceptual clarity, emotional symptoms, including anxiety, depression, and distress, were classified as psychological, whereas memory impairment and attention deficits were categorized within the cognitive domain. When both psychological and cognitive symptoms co-occurred in a cluster, they were explicitly labeled as mixed psychological–cognitive clusters.

Although the majority of symptom clusters centered on physiological and psychological dimensions, several studies identified clusters involving social withdrawal, role functioning impairments, or other behavioral changes. These were grouped under a distinct category termed social function–related clusters, in recognition of their clinical importance and potential impact on quality of life.

Symptom incidence rates varied considerably across studies. For instance, nausea and appetite loss were reported in approximately 20% to 70% of patients, while distress, fatigue, and sleep disturbances occurred in 15% to 60%, depending on the timing of assessment and measurement tools used.

### 3.5 Temporal evolution of symptom clusters across chemotherapy stages

**Figure 2:**
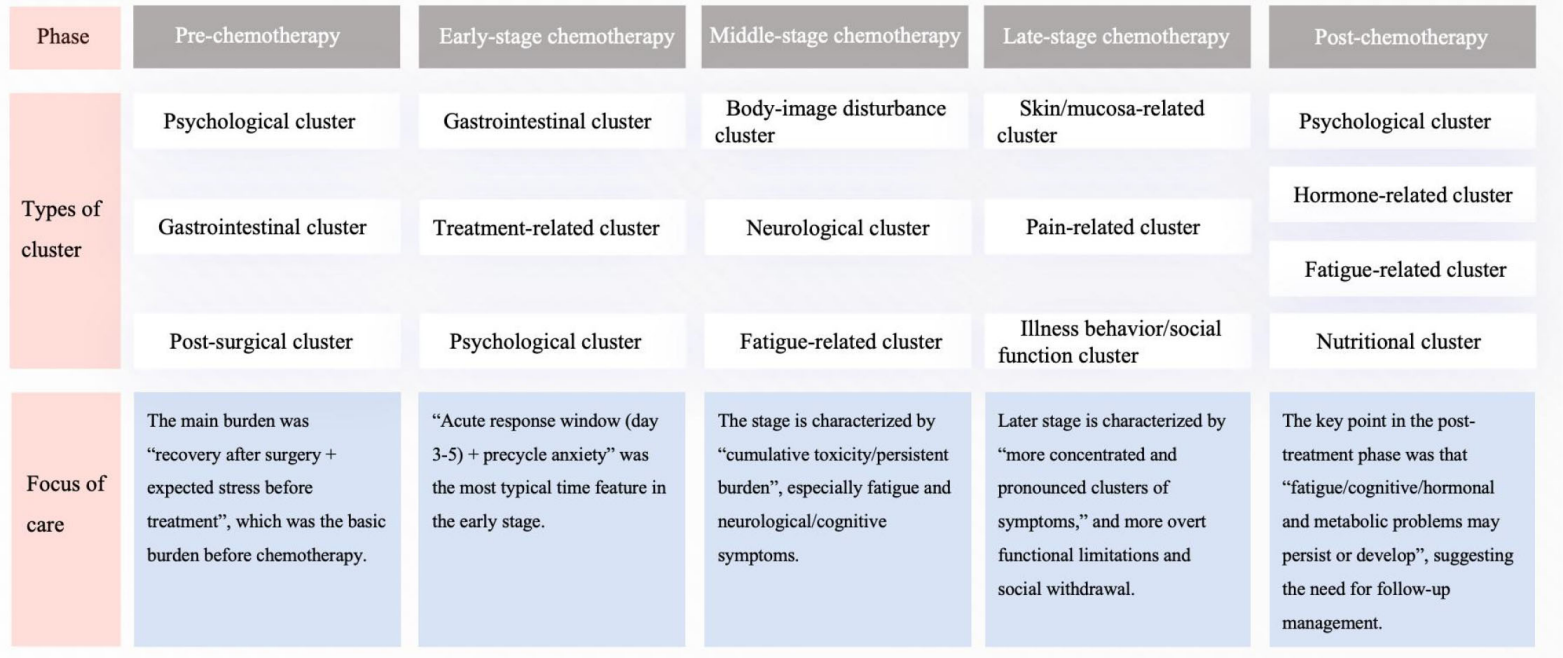
Symptom cluster composition and clinical care focus across chemotheraphy phases in breast cancer patients.

To illustrate symptom cluster dynamics, a chemotherapy phase-based framework was applied NCCN guidelines (2023): pre-chemotherapy, during chemotherapy (early stage: 1–2 weeks/first cycle, middle stage: 3–6 weeks/second- third cycles, and late stage: ≥6 weeks/after the fourth cycle), and post-chemotherapy. Through systematic analysis, clusters reported in ≥3 studies at the same time point were classified as typical symptom clusters. **Figure 2** presents a comprehensive overview of the various symptom cluster types and the primary focus of clinical care across different treatment phases.

#### 3.5.1 Pre-chemotherapy

Breast cancer patients exhibit three characteristic symptom clusters prior to chemotherapy initiation (20, 22–27, 29, 30):

- Psychological cluster: anxiety and depression, as well as anticipatory concerns about the side effects of the treatment.
- Gastrointestinal cluster: paradoxical bowel dysfunction, such as constipation or diarrhea.
- Post-surgical cluster: incision pain, lymphedema, and restricted limb mobility.

#### 3.5.2 Early-stage chemotherapy

Core symptom clusters demonstrate temporal intensification patterns (10, 11, 14–16, 18, 21, 23, 24, 28–31, 33):

- Gastrointestinal cluster: peak nausea and appetite loss (day 3–5 post-infusion).
- Treatment-related cluster: bone marrow suppression (neutrophils and white blood cells decreasing), immune weakening.
- Psychological cluster: acute emotional distress pre-cycle.

#### 3.5.3 Middle-stage chemotherapy

Compared with the early stage, the typical symptom clusters in the mid-stage of chemotherapy include (12, 13, 15, 17, 19–23, 25, 27, 29, 31, 33):

- Body-image disturbance cluster: hair loss, body image issues.
- Neurological cluster: paresthesia, dizziness, cognitive impairment symptoms.
- Fatigue-related cluster: persistent exhaustion, often anemia-related.

#### 3.5.4 Late-stage chemotherapy

At this stage, the patients show a clustering phenomenon, and the typical symptom clusters are more prominent compared to those in the middle stage (15, 19–23, 26–33):

- Skin/mucosa-related cluster: stomatitis, dry skin.
- Pain-related cluster: joint/muscle pain.
- Illness behavior/social function cluster: social withdrawal, reduced mobility.

#### 3.5.5 Post-chemotherapy

After therapy, gastrointestinal clusters (such as nausea and decreased appetite) largely resolved; however, several new or persistent clusters emerged in the post-treatment period (22, 26, 28):

- Psychological cluster: “chemo brain”, residual anxiety.
- Hormone-related cluster: amenorrhea, hot flashes, libido changes.
- Fatigue-related cluster: 20% patients meet chronic fatigue criteria at 6 months.
- Nutritional cluster: appetite normalization delay, metabolic imbalance.

## 4 Discussions

### 4.1 Dynamic changes in symptom clusters and treatment approaches during chemotherapy for breast cancer

Following chemotherapy for breast cancer, patients’ symptom clusters exhibit dynamic changes that impact functional status and quality of life, consistent with established frameworks such as the Miaskowski Symptom Cluster Framework. This taxonomy classifies clusters based on stable co-occurrence, inter-symptom correlation, and shared impact on function and quality of life, as demonstrated in women undergoing chemotherapy (34). Anxiety, insomnia, and cognitive complaints are among the most common psychological-cognitive symptoms reported prior to chemotherapy (22), often driven by concerns regarding prognosis and upcoming treatment (35). Therefore, healthcare professionals may consider using validated instruments such as the Self-Rating Depression Scale (SDS) and the Self-Rating Anxiety Scale (SAS) to screen for psychological distress, particularly when patients report prominent anxiety or sleep disturbance. Early identification is important because psychological symptoms are closely correlated with fatigue and sleep disturbance, amplifying overall symptom burden (36). In addition, emerging evidence indicates that structured cognitive stimulation interventions may alleviate chemotherapy-related cognitive impairment in breast cancer patients (37). Accordingly, a comprehensive pre-chemotherapy assessment of mental health and cognition together with timely, individualized support may help reduce symptom severity and improve treatment tolerance.

As chemotherapy progresses, symptom clusters form and intensity changes considerably. Along with worry and fatigue, patients typically experience significant gastrointestinal symptoms during the first week of chemotherapy (24, 25). Nurses can evaluate gastrointestinal symptoms, develop customized care plans, and adjust patients’ dietary regimens using the Edmonton Symptom Assessment System (ESAS) and the MD Anderson Symptom Inventory (MDASI) (36). The degree of fatigue can also be measured using the Multidimensional Fatigue Symptom Inventory-Short Form (MFSI-SF), which aids in the early detection of symptom changes and timely intervention (38).

After the second week of chemotherapy, gastrointestinal difficulties may disappear, but psychological problems may get worse (26). In addition to providing individualized solutions and support, nurses should now pay greater attention to their patients’ psychological health. Many patients have issues with their self-image by the third month, such as weight swings and hair loss, which significantly affects their mental health (22, 24). Furthermore, gastrointestinal issues, including nausea, vomiting, and appetite loss, as well as fatigue symptoms like sluggishness and general weakness, may get worse. These symptoms are often associated with the cumulative side effects of chemotherapy drugs (39). During this stage, nursing personnel should focus on assessing patients’ physical symptoms and closely monitor any changes so that the treatment plan may be quickly adjusted.

### 4.2 Targeted nursing interventions for patients with breast cancer who experience dynamic core symptoms during chemotherapy

This study found that symptom clusters in breast cancer patients change dynamically as chemotherapy advances, requiring phase-specific nursing interventions. Clinically, this implies that a single, static symptom management plan is unlikely to address the shifting symptom burden across cycles. Therefore, nursing care should be organized around standardized trajectory anchors and integrated with routine symptom monitoring to enable timely anticipatory interventions. In the pre-treatment and early stages, anxiety and appearance-related concerns are particularly prominent (21). According to research, anxiety can be reduced through relaxation techniques such as progressive muscle relaxation and controlled abdominal breathing (40, 41). In addition, frequent psychological counseling or group-based support may incorporate mindfulness meditation and cognitive behavioral therapy (CBT) to help patients manage anxiety and distress (42). Progressive muscle relaxation training can begin one week prior to treatment, and the optimal frequency and timing remain uncertain; these approaches may enhance psychological resilience and alleviate anxiety and depressive symptoms (43). During treatment breaks, nurses may also organize group activities that allow patients to share experiences and provide peer support, thereby strengthening coping skills and self-efficacy. In addition, ongoing psychological care should be provided throughout chemotherapy to help patients maintain emotional well-being.

Gastrointestinal symptoms, including nausea, vomiting, and appetite loss, are most prevalent during chemotherapy and have a substantial impact on patients’ physical strength and nutritional intake (22). To reduce digestive problems and increase physical strength, dietitians can create personalized diet regimens that include foods high in protein, vitamins, and digestibility, such as fish, lean meats, lentils, and fresh vegetables (44). Depending on their patients’ physical characteristics and taste preferences, dietitians can also provide tailored dietary advice. For example, avoiding fatty meals 24 hours prior to chemotherapy may help prevent nausea and vomiting (40).

The primary adverse effects of chemotherapy in the later stages are pain and exhaustion (22). An efficient method for reducing weariness and enhancing the quality of sleep is exercise or physical activity intervention (41). According to studies, moderate exercise may reduce fatigue by increasing blood circulation, producing endorphins, and strengthening muscles (42, 43). Exercise therapy may be beneficial for reducing fatigue and improving sleep quality (45, 46). Individualized, moderate-intensity activity (e.g., walking or yoga) can be considered based on the patient’s condition and safety profile, with ongoing monitoring and adjustment by nurses. Adjunct physical modalities (e.g., massage, heat/cold application) may provide symptomatic relief, although responses can vary (47).

Clusters of physical symptoms continue during treatment. Breathing difficulties and lightheadedness are the primary symptoms prior to treatment (30, 31). Nurses should routinely assess the rhythm, depth, and rate of a patient’s breathing in order to assess the severity of their breathing problems. To improve lung capacity and ease breathing, patients are recommended to assume a sitting or semi-recumbent position (48). Maintaining a healthy diet, getting enough sleep, and getting enough rest can also prevent dizziness (45). One of the main symptoms in the early phases of chemotherapy is hair loss. Counseling and psychological support are essential at this point. Modern medical technology has made it possible to lessen hair loss by using scalp cooling techniques prior to chemotherapy (46). The most common side effects during the intermediate stages of chemotherapy include dry mouth and oral ulcers. It is recommended that patients practice proper oral hygiene, which includes using a soft-bristle toothbrush, fluoride toothpaste, and saline or sodium bicarbonate solution to clean their mouths and lessen the discomfort of ulcers (49). Although issues related to sexual health and intimacy have been reported in other studies of patients receiving chemotherapy, these symptoms were not identified as a distinct symptom cluster in the present results and are therefore more appropriately regarded as a potential topic for future research rather than a current clinical focus in this discussion.

A multidisciplinary team of physicians, nurses, nutritionists, psychologists, and physical therapists is essential to managing symptoms during treatment. Physical therapists provide exercise regimens that are appropriate for each patient, psychologists provide psychological support, and dietitians offer personalized food recommendations (50). To ensure comprehensive care support, it is advised to schedule frequent multidisciplinary sessions to talk about treatment progress and symptom changes.

### 4.3 Individual differences in symptom clusters and the impact of chemotherapy regimens

Age, cultural background, psychological state, and chemotherapy regimen characteristics are the main factors influencing significant individual variability in symptom clusters (17, 18, 27). According to research, elderly individuals are more susceptible to physical discomfort because of deteriorating physiological processes, concomitant chronic conditions, and decreased treatment tolerance (51, 52). Younger patients, on the other hand, are more worried about problems related to their self-image, which have a bigger effect on their mental health. Cultural factors may also shape symptom perception and reporting, cross-country studies show variations in physical, emotional, and cognitive symptoms linked to illness beliefs and stigma around visible effects like alopecia, though validated multilingual tools like the MDASI enable consistent core symptom assessment across groups (22, 53). In order to give tailored nursing support, nurses must thus be aware of the variations in symptom clusters across various populations in clinical practice. Chemotherapy regimens strongly impact the development and intensity of symptom clusters. Severe symptom clusters often emerge during specific treatment phases due to cumulative drug toxicity. For instance, research by Liu Shuying et al. revealed that chemotherapy regimens that contain taxane medications, such as docetaxel and paclitaxel, are more likely to cause clusters of neurologic symptoms, such as limb pain, tingling, or numbness (17). Emerging evidence supports cryotherapy interventions such as frozen gloves and socks worn during infusions, which have demonstrated 40-60% reductions in taxane-induced peripheral neuropathy incidence and severity in randomized trials. Platinum-based medications frequently administered across multiple cycles, commonly trigger gastrointestinal symptom clusters alongside renal and cardiac risks, with peak severity often occurring mid-treatment (36, 54). These phase-specific clusters can worsen patients’ psychological condition and quality of life beyond physical effects.

The age, health, psychological condition, drug tolerance, cultural context, and socioeconomic status (SES) of patients must all be considered when designing chemotherapy regimens to lower symptom cluster occurrence and enhance quality of life (55). SES further influences social functioning clusters, with lower SES patients experiencing greater social isolation and role limitations due to treatment barriers, caregiving burdens, and financial toxicity, while higher SES correlates with stronger social support and workplace accommodations. Healthcare professionals should monitor symptom evolution closely, modify treatment plans promptly, and offer supportive care informed by social determinants of health. As precision medicine advances, personalized drug selection methods can predict patient responses, enabling optimal regimens that also account for socioeconomic barriers and minimize side effects.

### 4.4 Research on symptom clusters needs assessment tools to be standardized

There are notable variations in the evaluation instruments and symptom cluster selection across the included studies. Every widely used, globally standardized evaluation tool has advantages and disadvantages of its own.

- The Memorial Symptom Assessment Scale (MSAS) (56): It offers a thorough assessment of the frequency, intensity, and distress of symptoms throughout the previous seven days. Although it takes longer to administer and is less sensitive to particular symptom clusters or sudden changes, it is appropriate for evaluating the overall symptom load in cancer patients.
- The Edmonton Symptom Assessment System (ESAS) (57): Nine common symptoms that have occurred in the last 24 hours can be quickly assessed for severity using it, which is quick and simple to use. Although it is appropriate for quick clinical evaluation, it frequently ignores the distress or functional significance of symptoms and is not very good at capturing the intricacy of symptom clusters.
- The MD Anderson Symptom Inventory (MDASI) (58): It evaluates how severe symptoms are and how they have affected day-to-day functioning throughout the last 24 hours. Although it has extra modules for particular cancer types in addition to basic symptoms, its 24-hour recall span makes it challenging to record long-term symptom trends and leaves out important information about the emotional or psychological components of symptom clusters.
- The purpose of the Multidimensional Fatigue Symptom Inventory-Short Form (MFSI-SF) (38): It is to assess fatigue levels and how they affect cognitive, emotional, and physical functioning. Although it does not address any co-occurring symptoms, it is appropriate for evaluating the primary complaint of exhaustion.
- The Hospital Anxiety and Depression Scale (HADS) (59): Although it offers a rapid and accurate assessment of psychological distress, it is less sensitive to other emotional symptoms like irritation or emotional instability. Nevertheless, it is extensively used to measure anxiety and depression.

These tool variations contribute to substantial methodological heterogeneity, precluding formal meta-analysis. A critical limitation is differences in recall periods, which affect symptom cluster stability and comparability: “24-hour tools” (ESAS, MDASI) capture acute fluctuations but underestimate chronic cluster patterns and longitudinal stability, while “7-day recall tools” (MSAS) better reflect stable co-occurring clusters but may miss peak acute severity during chemotherapy cycles. Additional sources include variations in cluster identification methods, patient demographics, chemotherapy regimens, and follow-up durations. Instead, narrative synthesis was employed to integrate findings thematically by chemotherapy phase and symptom trajectory patterns. Future reviews may benefit from evidence mapping to visualize evidence distribution across symptom clusters, populations, and recall periods.

Because there is currently no single standard for symptom cluster research, different assessment methods produce different outcomes. Scholars have proposed that new assessment tools should incorporate multidimensional assessment indicators to accommodate diverse patient groups (55, 60). These instruments can better capture symptom impact on quality of life through patient-reported descriptions and sentiments.

According to this study, breast cancer patients experience dynamic symptom cluster changes during chemotherapy. Dynamic monitoring systems (e.g., mobile apps for real-time symptom logging) and cross-cultural validation are needed to enhance clinical utility and global applicability (61–63). Standardized instruments can reduce variability in study results by offering a uniform framework for detecting and quantifying symptom clusters. This uniformity is essential for creating more trustworthy clinical guidelines and therapies as well as for comparing outcomes across various research and populations. In the end, this can increase quality of life, improve patient outcomes, and maximize the therapeutic experience for patients.

### 4.5 Future research directions and suggestions

The absence of accepted identification techniques and standardized evaluation instruments currently hinders the investigation of symptom clusters. Cross-study comparisons are difficult due to the substantial heterogeneity of research findings caused by this shortcoming. Future studies should concentrate on creating and evaluating more thorough, sophisticated evaluation instruments that incorporate patient self-assessment and clinician observations in order to more precisely identify symptom clusters and their dynamic changes. Additionally, more prompt and individualized clinical care intervention grounds can result from the development of real-time dynamic monitoring systems with wearable and mobile health technologies.

Future studies should focus more on creating customized treatment strategies because different patient populations have quite different symptom clusters. We can anticipate how patients will react to chemotherapy medications, optimize chemotherapy regimens, and lessen the frequency and intensity of symptom clusters by using precision medicine techniques like genetic testing and drug sensitivity studies (64). Furthermore, creating all-encompassing nursing interventions that take into account patients’ psychological moods, social support networks, and cultural backgrounds can greatly improve their quality of life.

Many medical professions, including psychiatry, oncology, nursing, and rehabilitation medicine, are involved in treating symptom clusters. Future studies should improve multidisciplinary collaboration, carry out thorough intervention studies, and leverage the capabilities of several fields. More thorough recommendations for clinical practice can be obtained by looking at the results of multimodal therapies, such as combining physical rehabilitation, nutritional support, and psychotherapy.

Relatively few studies currently monitor long-term symptom clusters following chemotherapy; instead, the majority of research focuses on changes in symptom clusters during treatment. In order to investigate the long-term effects and connections with the healing process, future research should improve follow-up studies of long-term symptom clusters in breast cancer patients after chemotherapy. Longitudinal follow-up offers better knowledge of the natural progression of symptom clusters and provides the scientific foundation for rehabilitative therapy and follow-up therapies.

## 5 Strengths and limitations

The review provides a scientific foundation for therapeutic symptom management by methodically summarizing the forms, traits, and dynamic changes of symptom clusters in patients with breast cancer undergoing chemotherapy. It covers psychological, physiological, and social aspects. In all, 24 studies with 5,234 patients were included, providing a wealth of information that can more fully represent the symptom clusters in patients with breast cancer undergoing chemotherapy. Furthermore, it highlights the dynamic and phased nature of symptom cluster changes at various treatment stages and provides specific recommendations for clinical nursing interventions. The included studies do have several limitations, though, such as notable variations in patient characteristics and evaluation instruments, which result in a high degree of heterogeneity in the findings and could compromise the consistency and generalizability of the conclusions. The review was unable to do a meta-analysis because of the heterogeneity of the included studies and the absence of quantitative data in some of them, which prevented more accurate quantitative comparisons of the incidence and severity of symptom clusters.

## 6 Conclusions

Breast cancer patients have a wide range of symptom cluster types and dynamic changes during chemotherapy, encompassing psychological, physiological, and social dimensions. These symptom clusters also exhibit distinct characteristics at different stages of chemotherapy. There is currently no agreement on assessment instruments and no single standard for the identification and categorization of symptom clusters. In order to successfully lessen the symptom burden and enhance patients’ quality of life, healthcare professionals should provide tailored therapies based on the primary symptom clusters at each stage of chemotherapy. The assessment instruments and techniques for symptom clusters should be further standardized in future studies to allow for more precise diagnosis and management in clinical nursing practice, leading to more scientific and useful nursing management plans for patients with breast cancer.

## Data Availability

The datasets presented in this study can be found in online repositories. The names of the repository/repositories and accession number(s) can be found in the article/**Supplementary Material**.
