# Supplementary material for: Symptom clusters and their temporal patterns in breast cancer patients undergoing chemotherapy: a systematic review of cross-sectional and longitudinal studies
Source: Front Oncol. 2026 Mar 16;16:1717693. doi: 10.3389/fonc.2026.1717693 (PMC13033555; doi:10.3389/fonc.2026.1717693)
Supplement: Supplementary file 1 [file Table1.docx]

**SUPPLEMENTARY DATA:**

Symptom Clusters and Their Temporal Patterns in Breast Cancer Patients Undergoing Chemotherapy: A Systematic Review of Cross-Sectional and Longitudinal Studies

Contents of Supplements

[Appendix 1 Full search strategy used in this systematic review 2](#_Toc119338440)

[Appendix 2 Extracted basic information from included studies 7](#_Toc119338441)

[Appendix 3 Kmet checklist 1](#_Toc119338442)0

[Appendix 4 Quality assessment of the included studies by Kmet checklist 1](#_Toc119338443)2

# **Appendix 1 Full search strategy used in this systematic review**

Database One: PubMed

| **Search number** | **Query** |
| --- | --- |
| **#1** | ((((((((((“Breast Neoplasms”[Mesh]) OR (Breast Neoplasm[Title/Abstract])) OR (Breast Tumor[Title/Abstract])) OR (Breast Cancer[Title/Abstract])) OR (Cancer of Breast[Title/Abstract])) OR (Malignant Neoplasm of Breast[Title/Abstract])) OR (Mammary Cancer[Title/Abstract])) OR (Mammary Neoplasm, Human[Title/Abstract])) OR (Breast Carcinoma[Title/Abstract])) OR (Mammary Carcinoma, Human[Title/Abstract])) |
| **#2** | ((((((“Syndrome”[Mesh]) OR (Symptom Cluster[Title/Abstract])) OR (Symptom constellation[Title/Abstract])) OR (Concurrent symptom[Title/Abstract])) OR (multiple symptom[Title/Abstract])) OR (symptom combination[Title/Abstract])) |
| **#3** | #1 AND #2 |

Database Two: Embase

| **Search number** | **Query** |
| --- | --- |
| **#1** | 'Breast Neoplasms':ti,ab,kw OR 'Breast Tumor':ti,ab,kw OR 'Breast Cancer':ti,ab,kw OR 'Cancer of Breast':ti,ab,kw OR 'Malignant Neoplasm of Breast':ti,ab,kw OR 'Mammary Cancer':ti,ab,kw OR 'Mammary Neoplasm, Human':ti,ab,kw OR 'Breast Carcinoma':ti,ab,kw OR 'Mammary Carcinoma, Human':ti,ab,kw |
| **#2** | 'Syndrome':ti,ab,kw OR 'Symptom Cluster':ti,ab,kw OR 'Symptom constellation':ti,ab,kw OR 'Concurrent symptom':ti,ab,kw OR 'multiple symptom':ti,ab,kw OR 'symptom combination':ti,ab,kw |
| **#3** | #1 AND #2 |

Database Three: Web of science

| **Search number** | **Query** |
| --- | --- |
| **#1** | TS=(Breast Neoplasms or Breast Tumor or Breast Cancer or Cancer of Breast or Malignant Neoplasm of Breast or Mammary Cancer or Mammary Neoplasm, Human or Breast Carcinoma or Mammary Carcinoma, Human) |
| **#2** | TS=(Syndrome or Symptom Cluster or Symptom constellation or Concurrent symptom or multiple symptom or symptom combination) |
| **#3** | #1 AND #2 |

Database Four: Cochrane Library

| **Search number** | **Query** |
| --- | --- |
| **#1** | (Breast Neoplasms):ti,ab,kw OR (Breast Tumor):ti,ab,kw OR (Breast Cancer):ti,ab,kw OR (Cancer of Breast):ti,ab,kw OR (Malignant Neoplasm of Breast):ti,ab,kw OR (Mammary Cancer):ti,ab,kw OR (Mammary Neoplasm, Human):ti,ab,kw OR (Breast Carcinoma):ti,ab,kw OR (Mammary Carcinoma, Human):ti,ab,kw |
| **#2** | (Syndrome):ti,ab,kw OR (Symptom Cluster):ti,ab,kw OR (Symptom constellation):ti,ab,kw OR (Concurrent symptom):ti,ab,kw OR (multiple symptom):ti,ab,kw OR (symptom combination):ti,ab,kw |
| **#3** | #1 AND #2 |

Database Five: VIP(维普数据知识服务平台)

| **Search number** | **Query** |
| --- | --- |
| **#1** | 题名或关键词:(乳腺肿瘤)+题名或关键词:(乳腺癌)+题名或关键词:(乳腺恶性肿瘤) |
| **#2** | 题名或关键词:(症状群)+题名或关键词:(症状组合)+题名或关键词:(并发症状)+题名或关键词:(多种症状) |
| **#3** | #1 AND #2 |

Database Six: Wanfang(万方数据知识服务平台)

| **Search number** | **Query** |
| --- | --- |
| **#1** | 题名或关键词:(乳腺肿瘤)+题名或关键词:(乳腺癌)+题名或关键词:(乳腺恶性肿瘤) |
| **#2** | 题名或关键词:(症状群)+题名或关键词:(症状组合)+题名或关键词:(并发症状)+题名或关键词:(多种症状) |
| **#3** | #1 AND #2 |

Database Seven: CNKI(中国知网)

| **Search number** | **Query** |
| --- | --- |
| **#1** | (TKA=乳腺肿瘤) OR (TKA=乳腺癌) OR (TKA=乳腺恶性肿瘤) |
| **#2** | (TKA=症状群) OR (TKA=症状组合) OR (TKA=并发症状) OR (TKA=多种症状) |
| **#3** | #1 AND #2 |

Database Eight: Sinomed

| **Search number** | **Query** |
| --- | --- |
| **#1** | “乳腺肿瘤”[常用字段:智能] OR “乳腺癌”[常用字段:智能] OR “乳腺恶性肿瘤”[常用字段:智能] |
| **#2** | “症状群”[常用字段:智能] OR “症状组合”[常用字段:智能] OR “并发症状”[常用字段:智能] OR “多发症状”[常用字段:智能] |
| **#3** | #1 AND #2 |

| Appendix 2.Extracted basic information from included studies | | | | | | |  |
| --- | --- | --- | --- | --- | --- | --- | --- |
| **Author** | **Year** | **Country** | **Study type** | **Sample size** | **Tumor stage** | **Chemotherapy medications** |  |
| Chongkham-Ang et al^[10]^ | 2018 | Thailand | Cross-sectional | 322 | I~III | 5 fluorouracil, doxorubicin, cyclophosphamide/doxorubicin |  |
| Vuttanon et al^[12]^ | 2019 | Thailand | Cross-sectional | 48 | Not reported | Fluorouracil, epirubicin, cyclophosphamide, docetaxel | |
| Langford et al^[13]^ | 2016 | American | Cross-sectional | 391 | 0~III | Not reported | |
| Deng et al^[14]^ | 2020 | China | Cross-sectional | 240 | I~III | Not reported | |
| Jiang et al^[15]^ | 2018 | China | Cross-sectional | 164 | Not reported | EC/AC/TA/CEF/THP, C/TE/T/TEC | |
| Li et al^[16]^ | 2021 | China | Cross-sectional | 154 | I~III | TAX, dexamethasone/diphenhydramine/cimetidine | |
| Li et al^[17]^ | 2020 | China | Cross-sectional | 218 | I~IV | Not reported | |
| Liu et al^[18]^ | 2015 | China | Cross-sectional | 65 | I~IV | Not reported | |
| Wang et al^[19]^ | 2015 | China | Cross-sectional | 169 | I~III | Anthracycline, TAX | |
| Zhang et al^[20]^ | 2017 | China | Cross-sectional | 128 | I~IV | Anthracycline, TAX | |
| Liang et al^[21]^ | 2024 | China | Cross-sectional | 468 | I~III | FEC 75/FEC 100, docetaxel | |
| Zhou et al^[22]^ | 2016 | China | Cross-sectional | 296 | I~III | Not reported | |
| Albusoul et al^[23]^ | 2017 | American | Longitudinal | 219 | I~IIIa | TAX | |
| Browall et al^[24]^ | 2017 | Sweden | Longitudinal | 124 | I~IIIa | FEC 75/FEC 100, docetaxel | |
| Sullivan et al^[25]^ | 2018 | American | Longitudinal | 540 | I~III | doxorubicin/cyclophosphamide/TAX/docetaxel/fluorouracil | |
| Golan-Vered et al^[26]^ | 2013 | Lsrael | Longitudinal | 40 | I~III | Doxorubicin, cyclophosphamide, TAX | |
| Berger et al^[27]^ | 2020 | American | Longitudinal | 202 | Not reported | Anthracycline/TAX | |
| Sanford et al^[28]^ | 2014 | American | Longitudinal | 80 | Not reported | Not reported | |
| Huang et al^[29]^ | 2024 | China | Longitudinal | 108 | Not reported | Not reported | |
| Li et al^[30]^ | 2016 | China | Longitudinal | 215 | I~IV | Carboplatin combination | |
| Zhu et al^[31]^ | 2022 | China | Longitudinal | 200 | I~III | Anthracycline, TAX, cyclophosphamide/TAX, cyclophosphamide/TAX, platinum/Anthracycline, cyclophosphamide | |
| Luo et al^[32]^ | 2023 | China | Longitudinal | 620 | I~III | TAC/AC-T/EC-T/TEC/TA/TCb | |
| Hsin-Tien Hsu et al^[33]^ | 2017 | China | Longitudinal | 103 | 0~III | Cyclophosphamide, epirubicin, fluorouracil | |
| Wang et al^[34]^ | 2023 | China | Longitudinal | 120 | I~IV | Not reported | |

**Appendix 3 Kmet checklist**

| **No.** | **Evaluation Item** | **Score (0-2)** |
| --- | --- | --- |
| 1 | Question/objective sufficiently described? |  |
| 2 | Study design evident and appropriate? |  |
| 3 | Method of subject/comparison group selection or source of information/input variables described and appropriate? |  |
| 4 | Subject (and comparison group, if applicable) characteristics sufficiently described? |  |
| 5 | If interventional and random allocation was possible, was it described? (Only applicable to RCTs) | N/A |
| 6 | If interventional and blinding of investigators was possible, was it reported? (Only for RCTs) | N/A |
| 7 | If interventional and blinding of subjects was possible, was it reported? (Only for RCTs) | N/A |
| 8 | Outcome and exposure measures well defined and robust to measurement/misclassification bias? Means of assessment reported? |  |
| 9 | Sample size appropriate? (May be N/A for exploratory studies) |  |
| 10 | Analytic methods described/justified and appropriate? |  |
| 11 | Some estimate of variance is reported for the main results? |  |
| 12 | Controlled for confounding? (May be N/A for descriptive/exploratory studies) | N/A |
| 13 | Results reported in sufficient detail? |  |
| 14 | Conclusions supported by the results? |  |

Note:The methodological quality of each included study was assessed using the 14-item checklist developed by Kmet et al. (2004) for evaluating primary research papers in quantitative studies. Each item was scored on a 3-point scale (Yes = 2, Partial = 1, No = 0), with non-applicable items (e.g., items 5–7, 12) excluded from the total score. Final quality scores were calculated as a percentage and used to categorize studies as limited (<50%), adequate (50–69%), good (70–79%), or strong (≥80%).

| Appendix 4.Quality assessment of the included studies by Kmet checklist | | | | | | | | | | | | | | | |
| --- | --- | --- | --- | --- | --- | --- | --- | --- | --- | --- | --- | --- | --- | --- | --- |
| **Study/year** | **Item 1** | **Item 2** | **Item 3** | **Item 4** | **Item 5** | **Item 6** | **Item 7** | **Item 8** | **Item 9** | **Item 10** | **Item 11** | **Item 12** | **Item 13** | **Item 14** | **Quality score(%)** |
| Chongkham-Ang et al (2018) | 2 | 2 | 2 | 2 | N/A | 1 | 2 | 1 | 1 | 2 | 2 | 2 | 2 | 1 | 22(85%) |
| Vuttanon et al (2019) | 2 | 2 | 2 | 2 | 1 | 0 | 0 | 2 | 1 | 2 | 1 | 1 | 2 | 2 | 20(71%) |
| Langford et al (2016) | 2 | 2 | 2 | 2 | 1 | 1 | 2 | 2 | 1 | 2 | 2 | 2 | 2 | 1 | 24(86%) |
| Zhenzhen,Deng et al (2020) | 2 | 2 | 2 | 1 | N/A | 1 | 2 | 1 | 1 | 2 | 2 | 2 | 2 | 1 | 21(81%) |
| Lei Jiang et al (2018) | 2 | 1 | 1 | 1 | N/A | 1 | 2 | 1 | 1 | 2 | 2 | 2 | 1 | 1 | 18(69%) |
| Li yan,Li et al (2021) | 2 | 2 | 1 | 1 | N/A | 1 | 2 | 1 | 1 | 2 | 2 | 2 | 2 | 1 | 20(77%) |
| Xiao feng,Li et al (2020) | 2 | 2 | 1 | 1 | N/A | 1 | 2 | 1 | 1 | 2 | 2 | 2 | 1 | 1 | 19(73%) |
| Shu ying,Liu et al (2015) | 2 | 2 | 1 | 1 | N/A | 1 | 2 | 1 | 1 | 2 | 2 | 2 | 2 | 1 | 20(77%) |
| Qian xin,Wang et al (2015) | 2 | 2 | 2 | 2 | N/A | 1 | 2 | 2 | 1 | 2 | 2 | 2 | 1 | 1 | 22(85%) |
| Jing Zhang et al (2017) | 2 | 2 | 2 | 2 | N/A | 1 | 2 | 2 | 1 | 2 | 2 | 2 | 2 | 1 | 23(88%) |
| Minyu,Liang et al (2024) | 2 | 1 | 2 | 1 | 2 | 2 | 2 | 2 | 1 | 2 | 2 | 2 | 2 | 2 | 25(89%) |
| Chun lan,Zhou et al (2016) | 2 | 2 | 1 | 1 | N/A | 1 | 2 | 1 | 1 | 2 | 2 | 2 | 2 | 1 | 20(77%) |
| Albusoul et al (2017) | 2 | 2 | 1 | 2 | N/A | N/A | N/A | 2 | 2 | 2 | 1 | 1 | 2 | 2 | 19(86%) |
| Maria Browall et al (2017) | 2 | 2 | 2 | 1 | 1 | 2 | 2 | 1 | 2 | 2 | 2 | 2 | 2 | 1 | 24(86%) |
| Carmen W. Sullivan et al (2018) | 2 | 2 | 2 | 2 | N/A | N/A | N/A | 2 | 2 | 2 | 1 | 1 | 2 | 2 | 20(91%) |
| Golan-Vered et al (2013) | 2 | 1 | 2 | 1 | 1 | 2 | 2 | 1 | 1 | 1 | 2 | 2 | 1 | 1 | 20(71%) |
| Berger et al (2020) | 2 | 2 | 1 | 2 | N/A | N/A | N/A | 2 | 2 | 2 | 2 | 1 | 2 | 2 | 20(91%) |
| SanFord et al (2014) | 2 | 2 | 2 | 2 | N/A | N/A | N/A | 2 | 1 | 2 | 2 | 1 | 2 | 2 | 20(91%) |
| Xue fei,Huang et al (2024) | 2 | 1 | 2 | 1 | 2 | 1 | 2 | 1 | 1 | 1 | 1 | 1 | 1 | 1 | 18(64%) |
| Nan nan,Li et al (2016) | 2 | 2 | 1 | 1 | 2 | 1 | 2 | 1 | 1 | 2 | 2 | 2 | 2 | 1 | 22(79%) |
| Long lin,Zhu et al (2022) | 2 | 2 | 2 | 2 | 2 | 1 | 2 | 1 | 1 | 2 | 2 | 2 | 2 | 1 | 24(86%) |
| Luo et al (2023) | 2 | 2 | 2 | 2 | 1 | 1 | 2 | 1 | 2 | 2 | 2 | 2 | 2 | 1 | 24(86%) |
| Hsin-Tien Hsu et al (2017) | 2 | 2 | 2 | 2 | 1 | 1 | 2 | 1 | 2 | 2 | 2 | 2 | 2 | 1 | 24(86%) |
| Wang et al (2023) | 2 | 2 | 2 | 2 | 1 | 1 | 2 | 1 | 1 | 2 | 2 | 2 | 2 | 1 | 23(8%) |
